# Controversies in the Adjuvant Therapy of Endometrial Cancer

**DOI:** 10.5402/2011/724649

**Published:** 2011-09-29

**Authors:** Sheng-Mou Hsiao, Lin-Hung Wei

**Affiliations:** ^1^Department of Obstetrics and Gynecology, Far Eastern Memorial Hospital, Banqiao, New Taipei 220, Taiwan; ^2^Department of Obstetrics and Gynecology, National Taiwan University College of Medicine and National Taiwan University Hospital, Taipei 100, Taiwan; ^3^Department of Oncology, National Taiwan University College of Medicine and National Taiwan University Hospital, Taipei 100, Taiwan

## Abstract

Endometrial cancer is the most common malignancy of the female genital tract. Surgical treatment includes hysterectomy, bilateral salpingo-oophorectomy, and an appropriate staging procedure. Relapse of endometrial cancer may occur in patients with high risk factors, such as old age, grade 3 cancer, deep myometrial invasion, and papillary serous and clear cell types. In recent years, several randomized trials reported the results of adjuvant therapy for patients with high risk factors. Nonetheless, some controversies still exist. This paper presents and discusses the results of important randomized trials of adjuvant therapy for endometrial cancer with risk factors.

## 1. Introduction

Endometrial cancer (EC) is the most common malignancy of the female genital tract in the Western world with an annual incidence of 15–18/100,000 women [1]. Around 80% of EC patients is diagnosed in the early period of the disease and has a good prognosis. Surgical treatment, including hysterectomy, bilateral salpingo-oophorectomy, and an appropriate surgical staging workup. should be performed in all patients with EC. For better communication of the text below, the following descriptions of previous randomized trials are based on the International Federation of Gynecology and Obstetrics (FIGO) 1988 classification system instead of the FIGO 2009 staging system. Nonetheless, it is noteworthy to understand that stage IA and stage IB of the FIGO 1988 staging system are merged as stage IA of FIGO 2009 staging system; stage IIA is merged with stage I; positive cytology has to be reported separately without changing the stage; stage IIIC is subdivided into stage IIIC1 (no para-aortic lymph node metastasis) and stage IIIC2 (with para-aortic lymph node metastasis) [2].

Based on the National Comprehensive Cancer Network (NCCN) guidelines, observation is recommended for stage IA, grade 1 (G1) or grade 2 (G2) and stage IB, G1 patients with no adverse prognostic factors [3]. Röper et al. reported the major prognostic factors for early-stage EC to include older age, histologic type (i.e., serous or clear cell type), high histologic grade, deep myometrial invasion, lymphovascular space invasion (LVSI), large tumor size (>2 cm), and involvement of the lower uterine segment or cervix [4]. The indication of adjuvant chemotherapy or radiotherapy is based on risk factors of recurrence. Nowadays, controversies still exist about postoperative adjuvant chemotherapy or radiotherapy for early EC with adverse prognostic factors and for advanced EC.

It is suggested that all women with FIGO stage IB, IC, IIA (occult: cervical extension without clinical evidence of cervical enlargement), and IIB (occult) and no evidence of lymph node involvement should be considered as members of the intermediate risk group [5]. The Gynecologic Oncology Group (GOG) 99 trial [6] further defined high-intermediate and low-intermediate risk groups from the GOG 33 [5]. High-intermediate risk is defined as: (1) at least 70 years of age with only one of the other risk factor (i.e., moderate to poorly differentiated tumor grade, presence of LVSI, and deep (>2/3) myometrial invasion), (2) at least 50 years of age with any two of the other risk factors, or (3) any age with all three of the other risk factors. All other patients in the intermediate risk group are defined as being in the low-intermediate risk group [6]. High risk is defined as having clear or serous cell type, gross involvement of the cervix (gross stage II), stage III, or stage IV. It must be noted that most trials did not enroll the pure high-intermediate risk or high risk patients for randomization. Nonetheless, we attempt to discuss adjuvant therapy from the perspectives of different risk factor subgroups by analyzing the results of the randomized trials.

## 2. Adjuvant Therapy for Low and Low-Intermediate Risk Groups of EC Patients

The risk of locoregional recurrence for EC patients with G1and G2 endometrioid cell type and superficial (<50%) myometrial invasion is about 5% or less [7, 8]. There is no evidence of benefit to support the use of adjuvant therapy for low and low-intermediate risk groups; therefore, these patients can be safely treated by surgery only [8], despite vaginal brachytherapy (VBT) use for adjuvant treatment for low-intermediate risk patients [4]. Besides, it was concluded that postoperative radiotherapy is not indicated in stage I patients <60 years of age and patients with G2 tumors with superficial invasion from the result of the Post-Operative Radiation Therapy in Endometrial Carcinoma (PORTEC)-1 trial (external beam radiotherapy 46 Gy versus observation) [9].

## 3. Adjuvant Therapy for the High-Intermediate Risk Group of EC Patients

According to NCCN guidelines, external beam radiotherapy (EBRT), VBT, or both, or observation can be performed for high-intermediate risk patients [2]. In the PORTEC-1 trial, patients with stage I (G1 with outer-half myometrial invasion, G2 with any invasion, or grade 3 with superficial myometrial invasion) were randomized to pelvic EBRT (46 Gy) or no further treatment after surgery. Postoperative EBRT for stage I patients reduced locoregional recurrence but had no impact on overall survival (OS) [9, 10]. Additionally, EBRT increased treatment-related morbidity [9]. In the GOG 99 trial, adjuvant EBRT (50.4 Gy) for stage IB, IC, and II (occult) patients had a substantial positive impact on 2-year cumulative recurrence (12% in the observation group and 3% in the EBRT group, *P* = 0.007), especially in the high-intermediate risk group. The estimated 4-year OS did not differ between the observation and the EBRT groups (86% versus 92%, *P* = 0.56, resp.) [6]. In the ASTEC/EN.5 trial, patients with high-intermediate and high risk (including stage IA and IB grade 3, IC of all grades, and serous or clear cell histology) were randomized to receive postoperative adjuvant EBRT (40–46 Gy) or observation. The results showed that EBRT could not be recommended as part of routine treatment for women with intermediate-risk or high-risk early-stage endometrial cancer with the aim of improving survival [11]. The absolute benefit of EBRT in preventing isolated local recurrence is small and is not without toxicity [11]. 

In the PORTEC-2 trial, patients with high-intermediate risk (age >60 years and stage IC G1 or G2 disease, stage IB grade 3 disease, or any age and stage IIA disease) were randomized to receive pelvic EBRT (46 Gy) or VBT (21 Gy high-dose rate or 30 Gy low-dose rate). Five-year locoregional recurrence rate (EBRT versus VBT = 2.1% versus 5.1%, *P* = 0.17), OS (79.6% versus 84.8%, *P* = 0.57) or disease-free survival (DFS, 78.1% versus 82.7%, *P* = 0.74) did not differ between these two groups. It was concluded that VBT is effective in ensuring vaginal control and a better quality of life, with fewer gastrointestinal toxic effects than with EBRT. Accordingly, VBT should be the adjuvant treatment of choice for patients with high-intermediate risk of recurrence [12, 13]. Nonetheless, is there any role for EBRT? Kong et al. reported that in stage I patients with multiple high risk factors, including stage IC and grade 3 (G3), the risk ratio of EC-related death is 0.65 (95% confidence interval 0.38 to 1.14, *P* = 0.13) after adjuvant EBRT [14]. 

In addition, is there any role for adjuvant chemotherapy (CT) for high intermediate risk patients? In a Finnish trial, patients with stages IA-IB G3 (*n* = 28) or IC–IIIA grades 1–3 (*n* = 128) were randomized to receive pelvic EBRT (56 Gy) or chemoradiotherapy (EBRT combined with three courses of cisplatin-epirubicin-cyclophosphamide). The addition of CT failed to improve OS or the recurrence rate, but appeared to increase bowel complications [1]. In the Japanese Gynecologic Oncology Group (JGOG) 2003 randomized trial, stage IC–IIIC patients with deep (≥50%) myometrial invasion and <75 years of age received adjuvant pelvic RT (greater than 90% of patients received 45–50 Gy EBRT only) or cyclophosphamide-doxorubicin-cisplatin for 3 or more courses [15]. A subgroup analysis revealed that the 5-year progression-free survival (PFS) rates in the RT and CT groups were 94.5% and 87.6%, resp. (*P* = 0.11); the 5-year OS rates were 95.1% and 90.8%, respectively (*P* = 0.28). However, among these 120 patients with stage IC, >70 years of age, or G3 endometrioid cell type or stage II or IIIA (positive cytology) with outer-half myometrial invasion, the CT group had significantly higher PFS rate (83.8% versus 66.2%, *P* = 0.02) and OS rate (89.7% versus 73.6%, *P* = 0.006) compared to that of the RT group [15]. Nonetheless, the above results were derived from post hoc analysis. Thus, the ideal adjuvant therapy for high-intermediate risk EC patients remain undetermined. An ongoing GOG 249 trial enrolled high risk stage I or II patients. These patients are randomized to receive pelvic RT (conventional or intensity-modulated EBRT with or without VBT) or VBT followed by 3 courses of carboplatin-paclitaxel [16].

## 4. Adjuvant Therapy for the High Risk Group of EC Patients

In an Italian trial, patients at high risk of recurrence (i.e., stage IC and G3, stage II and G3 with outer-half myometrial invasion, and stage III; two thirds of patients were stage III) were randomized to receive adjuvant CT (five courses of cisplatin-doxorubicin-cyclophosphamide) or EBRT (45–50 Gy). Survival did not differ between the two groups [17]. On the contrary, in the GOG 122 trial, patients with stage III or IV and <2 cm of postoperative residual tumor were randomized to receive doxorubicin-cisplatin or whole-abdominal irradiation (30 Gy, with an additional 15-Gy pelvic ± para-aortic boost). PFS and OS were better in the CT group compared that of the RT group [18]. Thus, adjuvant CT seemed not to be inferior to RT in the treatment of high risk patients. 

However, the issues of which CT regimen is the treatment of choice for patients with high risk and advanced EC remain undetermined. In the European Organization for Research and Treatment of Cancer (EORTC) 55872 trial, patients with advanced or recurrent EC were randomized to receive doxorubicin or cisplatin-doxorubicin treatment. The combined cisplatin-doxorubicin group had a higher response rate (43% versus 17%, *P* < 0.001) but no difference in OS (9 months versus 7 months, *P* = 0.065) compared with the doxorubicin group [19]. Sovak et al. reported that the carboplatin-paclitaxel regimen is a well-tolerated, active regimen for the treatment of resected stage III or IV high risk patients with a 3-year OS rate of 56%, the median time to progression of 13 months, and a median OS of 47 months [20]. In the GOG 177 trial, patients with stage III or IV, or recurrent EC were randomized to receive doxorubicin-cisplatin or doxorubicin-cisplatin-paclitaxel (with filgrastim support). The addition of paclitaxel to doxorubicin-cisplatin improved objective response (57% versus 34%, *P* < 0.01), PFS (median: 8.3 versus 5.3 months, *P* < 0.01), and OS (median: 15.3 versus 12.3 months, *P* = 0.037). However, it resulted in a higher rate of peripheral neuropathy [21]. There is an ongoing GOG 209 trial of patients with stage III or IV, or recurrent EC randomized to receive doxorubicin-cisplatin-paclitaxel (with filgrastim support) or carboplatin-paclitaxel for seven courses. The results of this ongoing trial may give us the answer of the appropriate chemotherapy regimen for the treatment of advanced EC [22]. 

Another question is whether the combined chemoradiotherapy is a better treatment choice for high risk EC patients. Klopp et al. reported stage IIIC patients treated without RT (EBRT ± VBT) had a high rate of locoregional recurrence. Additionally, relapse after RT for IIIC patients mainly occurred in G3 patients who may be likely to benefit from combined chemoradiotherapy [23]. In the EORTC 55991 trial (abstract only), patients with stages I, II, IIIA (positive cytology only), and IIIC (excluding para-aortic metastases) and clear, serous, and anaplastic cell types were enrolled. Most patients had two or more risk factors including G3, deep myometrial invasion, or DNA nondiploidy. Enrolled patients were randomized to RT (EBRT ± VBT) or combined chemoradiotherapy. The chemotherapy regimen before August 2004 was cisplatin-doxorubicin or -epirubicin; thereafter, it was changed to cisplatin-doxorubicin or -epirubicin, paclitaxel-epirubicin-carboplatin, or paclitaxel- carboplatin. The hazard ratio for PFS was 0.58 in favor of the combined chemoradiotherapy group (*P* = 0.046), and a 7% difference in estimated 5-year PFS was found [24]. There is an ongoing PORTEC-3 trial in which high-intermediate and high risk patients (stage IB with LVSI and G3, stage II and G3, stage IIIA or IIIC, and stage IB-III and serous or clear cell type) were randomized to pelvic EBRT (48.6 Gy) alone or concurrent chemoradiotherapy (EBRT and two courses of cisplatin) followed by adjuvant CT (carboplatin and paclitaxel for four courses). The results could tell us if the addition of concurrent and adjuvant CT to postoperative RT will increase 5-year OS and failure-free survival or not [25]. Additionally, there is an ongoing GOG 258 trial in which patients (stages I and II with serous or clear cell type and positive cytology, stage III-IVA) were randomized to receive carboplatin and paclitaxel for six courses or concurrent chemoradiotherapy (EBRT ± VBT and two courses of cisplatin) followed by four courses of carboplatin and paclitaxel [26].

In conclusion, the adjuvant treatment of choice for high-intermediate and high risk EC patients remains undetermined. Adjuvant therapy should be considered according to individual risk factors. Ongoing trials could resolve the controversies of adjuvant therapy in EC once they are completed.
